# UV-Induced Neuronal Degeneration in the Rat Cerebral Cortex

**DOI:** 10.1093/texcom/tgab006

**Published:** 2021-02-01

**Authors:** Mariko Nakata, Masayuki Shimoda, Shinya Yamamoto

**Affiliations:** 1 Integrative Neuroscience Research Group, Human Informatics and Interaction Research Institute, National Institute of Advanced Industrial Science and Technology (AIST), Tsukuba 305-8568, Japan; 2 Department of Pathology, Keio University School of Medicine, Tokyo 160-8582, Japan

**Keywords:** apoptosis, focal brain injury, focal brain lesion, neural degeneration, ultraviolet light

## Abstract

Irradiation with ultraviolet (UV) light on the cortical surface can induce a focal brain lesion (UV lesion) in rodents. In the present study, we investigated the process of establishing a UV lesion. Rats underwent UV irradiation (365-nm wavelength, 2.0 mWh) over the dura, and time-dependent changes in the cortical tissue were analyzed histologically. We found that the majority of neurons in the lesion started to degenerate within 24 h and the rest disappeared within 5 days after irradiation. UV-induced neuronal degeneration progressed in a layer-dependent manner. Moreover, UV-induced terminal deoxynucleotidyl transferase dUTP nick end labeling (TUNEL) positivity and heme oxygenase-1 (HO-1) immunoreactivity were also detected. These findings suggest that UV irradiation in the brain can induce gradual neural degeneration and oxidative stress. Importantly, UV vulnerability may vary among cortical layers. UV-induced cell death may be due to apoptosis; however, there remains a possibility that UV-irradiated cells were degenerated via processes other than apoptosis. The UV lesion technique will not only assist in investigating brain function at a targeted site but may also serve as a pathophysiological model of focal brain injury and/or neurodegenerative disorders.

## Introduction

Light is one of the most useful tools in modern neuroscience. It has been used in modulation and measurement of neural activity in vivo (Blasdel and Salama 1986; Deisseroth 2011; Gunaydin et al. 2014). Moreover, irradiation with excess amounts of light can induce destruction of the brain tissue (Suzuki et al. 2012; Nakata et al. 2018). For an effective use of light energy, it is necessary to investigate the underlying mechanisms of light-induced brain damage. Elucidating how light induces focal brain lesions could contribute in protecting neurons during optogenetic modulation and creating a focal brain lesion by light irradiation.

Generally, light with a shorter wavelength possesses higher energy but cannot travel a long distance within the biological tissue (Gebhart et al. 2006; Johansson 2010). Ultraviolet (UV) light has a wavelength of ~10–380 nm and is a powerful tool for neuroscientific manipulation. Previously, we developed a novel method for creating a focal brain lesion using UV light (365-nm wavelength), and we demonstrated that 365 nm is sufficiently long to reach the cortical surface through the dura (Nakata et al. 2018).

Previously, a variety of experimental methods for creating a focal brain lesion have been described, including electrical (Reynolds 1963; Kawakami et al. 1968) and pharmacological lesions (Schwarcz et al. 1979; Newsome et al. 1985) and lesions induced by physical damage (Ajika and Hökfelt 1975; Brownstein et al. 1975; Deuel and Mishkin 1977; Johnson et al. 2015), which requires insertion of an electrode or needle under the dura mater or is accompanied by dura break due to an impact. Compared with these methods, the UV lesion method is characteristic because the lesion is constantly shaped like an inverted bell, without drug spillover. The lesion size is quantitatively controllable by changing the amount of irradiation, and it does not require breakage of thedura.

The UV-lesion method enabled us to control the lesion size without demanding skill and was relieved from artifacts induced by dura mater breakage because of light permeability. However, the underlying mechanisms of lesion creation by UV irradiation are still unknown. Investigation of the process that establishes a UV lesion will enable us to use this technique not only for investigating brain function at a targeted site but also as a pathophysiological model of focal brain injury and/or neurodegenerative disorders.

UV irradiation induces tissue destruction, inflammation, DNA damage, and/or cell death when UV light is irradiated on the skin, eye, or hair (Buschke et al. 1945; Daniels Jr et al. 1961; Zi-Liang et al. 1990; Hoting et al. 1995; Kulms and Schwarz 2000; Ikehata et al. 2013; Ramasamy et al. 2017). On the other hand, little is known about the cellular and molecular effects of UV irradiation on the brain. In a previous study (Nakata et al. 2018), the effect of UV irradiation was examined 5 days (d) after the irradiation. It was reported that UV irradiation on the rodent cerebral cortex over the dura induced neural degeneration and glial congregation within and around an inverted bell-shaped UV lesion. In this study, we investigated the process that establishes UV-induced focal brain lesion. We histologically analyzed the time-dependent changes of the UV-irradiated site and the process of neural degeneration. Moreover, we intended to clarify the molecular basis of neural degeneration responding to UV irradiation on the cortical surface.

## Materials and Methods

### Experimental Animals

Adult male Wistar rats (total of *n* = 29; 332.66 ± 74.38 g body weight at the time of irradiation; purchased from SLC Japan Inc., Shizuoka, Japan) were used. All animals were housed under standard housing conditions (12-h light/dark cycle). Food and water were provided *ad libitum*. All procedures were conducted in accordance with the National Institutes of Health guidelines and were approved by the Animal Care and Use Committee of the National Institute of Advanced Industrial Science and Technology (AIST). All efforts were made to minimize the number of animals and their suffering. The following experimental protocols were determined in conformity to the previous study (Nakata et al. 2018).

### Irradiation with UV Light

Animals were anesthetized with an injection of ketamine (80 mg/kg, i.m.) and xylazine (10 mg/kg, i.p.) and placed in a stereotaxic frame (Narishige, Inc.). A constant level of anesthesia was maintained with 1% isoflurane. After removal of the skull bone, UV light from a UV-LED light source (LEDFLP-1CH_500, Doric Lenses, Inc.) was delivered through an optic cannula (MFC_400/430–0.37_12mm_SM3_FLT, Doric Lenses, Inc., 400-μm core diameter, 0.37 NA) contacting dura mater while avoiding blood vessels under the dura (anteroposterior −3.0 to −4.0 mm from bregma, mediolateral 1.5 to 2.5 from the midline). The intensity of the UV light was 1.0 mW in total (i.e., 7.96 mW/mm^2^ on average), which was measured at the tip of the optic cannula using a power meter (PM100D console with S130VC sensor, Thorlabs, Inc.; 365-nm wavelength). Animals received unilateral irradiation, in which the irradiated hemisphere was counter balanced and was exposed to 2.0 mWh (1.0 mW × 2 h, *n* = 22), 1.0 mWh (1.0 mW × 1 h, *n* = 3), or 4.0 mWh UV (1.0 mW × 4 h, *n* = 4). After 2.0 mWh of irradiation, the animals were transcardially perfused, at an interval of either 0 h (less than 15 min), 2 h, 6 h, 12 h, 24 h, and 3 d (*n* = 3 each, but one rat in the 3-d group was excluded from quantitative analysis due to thick vessel innervation at the center of the lesion), or 5 d (*n* = 4; but one rat was excluded from NeuN analysis because of failure of staining. To supplement the exclusion, one rat was included in NeuN analysis and HE stain). After the UV irradiation of 1.0 or 4.0 mWh, the animals were perfused at an interval of 5 d. Thereafter, brain samples were processed for subsequent histological analyses. Coronal sections of paraffin-embedded brain, sliced at 4-μm thickness, were generated using a sliding microtome. Then, sections were deparaffinized and subjected to hematoxylin and eosin (HE) staining or immunohistochemistry.

### Immunohistochemistry for Histological Analysis

Immunohistochemical staining for NeuN and glial fibrillary acidic protein (GFAP) was performed using a Bond-Max automated immunohistochemical staining machine (Leica Microsystems). The primary antibodies used were anti-NeuN (clone: A60, 1:100; Merck Millipore) and anti-GFAP (clone: 6F2, 1:200; DAKO A/S).

Immunohistochemical staining for ionized calcium-binding adapter molecule 1 (Iba1), heme oxygenase-1 (HO-1), NF-E2-related factor 2 (Nrf2), and myelin proteolipid protein (PLP) was performed as follows: deparaffinized sections on silane-coated glass slides were incubated in 0.02 M Tris–HCl buffer, pH 9.0 for 30 min at 90 °C. After cooling at room temperature (RT) and washing, the sections were incubated in methanol with 3% H_2_O_2_ for 20 min at RT for blocking endogenous peroxidase (POD) activity. After washing, sections were pretreated with PBS containing 0.2% Triton X (PBS–X) and 4% Block-Ace (blocking buffer; DS Pharma Biomedical Co., Ltd) for 2 h at RT. The sections were then incubated with goat polyclonal anti-Iba1 antiserum (1:100; ab104225, Abcam plc), rabbit monoclonal anti-HO-1 antiserum (1:50; ab68477, Abcam plc), rabbit polyclonal anti-Nrf2 antiserum (1:50; ab137550, Abcam plc), or rabbit polyclonal anti-myelin PLP antiserum (1:250; ab28486, Abcam plc) in blocking buffer overnight at either RT (Iba1 and HO-1), 38 °C (Nrf2), or 4 °C (PLP). Then, sections were washed and incubated with either biotinylated horse anti-goat secondary antiserum (1:250; BA-5000; Vector Laboratories, Inc.) for Iba1, or biotinylated goat anti-rabbit secondary antiserum (1:250; BA-1000; Vector Laboratories, Inc.) for HO-1, Nrf2, and PLP, for 2 h at RT. After washing, the sections were reacted with avidin-biotin complexes (VECTASTAIN Elite ABC Standard kit, PK-6100; Vector Laboratories, Inc.) in PBS for 1 h at RT and were then washed. Next, the sections were incubated in 0.02% diaminobenzidine (DAB) and 0.003% H_2_O_2_ containing 0.01 M imidazole for 5 min, followed by washing with PBS. All sections were counterstained with hematoxylin, dehydrated through an ascending ethanol series, cleared with xylene, and coverslipped with Permount (Thermo Fisher Scientific Inc.).

### TUNEL Staining and TUNEL-NeuN Double Staining

A commercially available kit for terminal deoxynucleotidyl transferase dUTP nick end labeling (TUNEL) apoptosis assay (In Situ Cell Death Detection Kit, POD, Roche Diagnostics International Ltd.) was used to detect single- and double-stranded DNA breaks at the early stages of apoptosis. The assay was conducted according to the manufacturer’s instructions. Briefly, deparaffinized sections were washed with PBS and were then permeabilized with 1.5% Proteinase K for 8 min at RT. After PBS washes, sections were immersed in 50 μL of TUNEL reaction mixture overnight at 4 °C. Sections were then washed with PBS and incubated with the Converter-POD reagent for 30 min at RT, followed by PBS washes. Finally, sections underwent DAB incubation, counterstaining, and dehydration as described above (see *Immunohistochemistry*). When double stained with TUNEL and NeuN, sections were washed with PBS-X after the DAB incubation, and they then underwent immunohistochemistry procedures for NeuN as described above (see *Immunohistochemistry*). For the double staining, NeuN-positive cells were colored with HistoGreen (Eurobio Ingen, Les Ulis), a commercially available substrate kit for POD instead of DAB incubation. Briefly, sections were incubated with a mixture of HistoGreen Chromogen, HistoGreen-Puffer and H_2_O_2_ for 5 min, followed by PBS washes and distilled water. Then, sections were dehydrated as described above (see *Immunohistochemistry*).

### Cell Counting

Sections containing the central area of the UV lesion were manually selected for histological analysis of immunopositive cells for NeuN and HO-1, and TUNEL-positive or -negative cells. The UV-lesioned area was photographed with a digital camera mounted to a microscope (microscope: Eclipse E100, Nikon Imaging Co. Ltd.; camera: Wi-Fi digital microscope camera HIS, Kenis Ltd.). The location of cells was manually marked using the image processing software ImageJ (National Institutes of Health). In particular, cells with strong TUNEL-positivity and morphology of dying cells at various stages are marked as “TUNEL-positive.” For cell marking, the region of interest (ROI) was set at the center of the lesion and dimensions of 200-μm width and 800-μm depth for HO-1 or either 800- or 1000-μm depth for NeuN (Supplementary Figure S1, gray line). When the lesion bottom was shallower than 800 μm (at 0 h, 2 h, and 5 d), the depth of the ROI in NeuN sections was set to 800 μm; otherwise, it was set to 1000 μm. Then, heatmaps indicating the distribution of NeuN- or HO-1-positive cells were created using a class as a depth and a degree as a cell number included in every 50-μm depth from the cortical surface using the R for Windows (version 3.2.2; R Core Team 2018).

**Figure 1:**
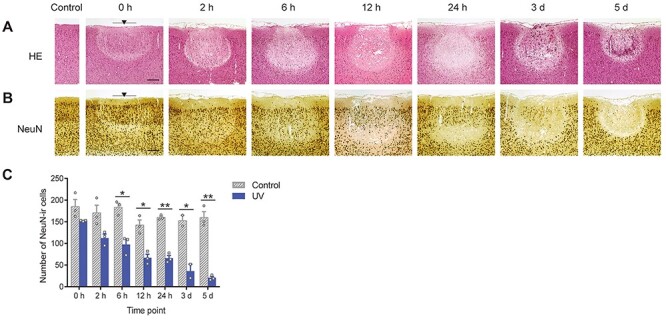
Neuronal degeneration process after UV irradiation. Representative photomicrographs of (*A*) HE-stained and (*B*) NeuN-immunostained (brown, neuronal marker) sections at each time point (0 h, 2 h, 6 h, 12 h, 24 h, 3 d, and 5 d) after UV irradiation (at bregma −3.84 mm). NeuN-immunostained sections were counterstained with hematoxylin (blue). Scale bar, 200 μm. Black arrowhead, center of the UV-lesioned site. Gray bar, coverage area of the core of the optic cannula (400 μm). These results were replicated at least 3 times with different animals each time. (*C*) The number of NeuN-positive cells gradually decreased after the irradiation at the UV-irradiated side (blue solid bars) but not at the control side (gray stripe bar, contralateral of the UV-irradiated side). Cells were counted within two ROIs of 200-μm square each and the total area of the ROIs was 0.08 mm^2^ (see *cell counting* in Materials and Methods). Each time point included 3 animals, except for 3 d, which included 2 animals. All data are presented as mean ± SEM. ^*^P < 0.05; ^**^P < 0.01.

For cell counting, top (layer II/III) and bottom (layers IV and V) ROIs with a 200-μm square each were set within the ROI (Supplementary Figure S1). The total amount of cell numbers included in top and bottom ROIs was used as the number of immunoreactive cells in Figures 1*C* (NeuN) and 2*C* (TUNEL). In this analysis, the total area of ROIs was 0.08 mm^2^. For NeuN-ir cell and TUNEL-positive counting, data were analyzed by a two-way analysis of variance (ANOVA), with main effects for time and repeated measurement of UV irradiation, and their interaction. Post hoc analysis was conducted with the Benjamini and Hochberg method for *P*-adjustment.

**Figure 2:**
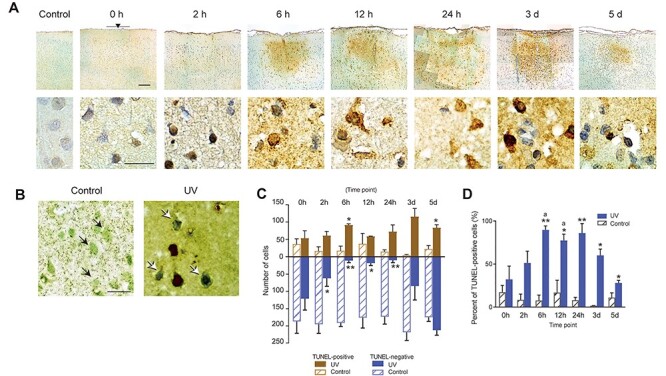
Apoptotic cell death after UV irradiation. (*A*) Representative photomicrographs of TUNEL-stained sections at each time point after UV irradiation (at bregma −3.84 mm). Brown: TUNEL-positive cells. Blue: cells counterstained with hematoxylin. Top panels: scale bar, 200 μm. Black arrowhead, center of the UV-lesioned site. Gray bar, coverage area of the core of the optic cannula (400 μm). Bottom panels: scale bar, 20 μm. (*B*) Representative photomicrographs of double-immunostained sections with TUNEL (brown) and NeuN (green). Both control (left panel) and UV-irradiated (right panel) sections were from the 6-h group. Black arrows indicate TUNEL-negative neurons. White arrows indicate TUNEL-positive neurons. Scale bar, 20 μm. These results were replicated at least 3 times with different animals each time. (*C*) The number of TUNEL-positive cells (brown bars) and TUNEL-negative, counterstained cells (blue bars) in the UV-irradiated (solid bars) and control (stripe bars) sides. (*D*) Percentage of TUNEL-positive cells in the total of TUNEL-positive and negative cells in the UV-irradiated (solid bars) and control (stripe bars) sides. Cells were counted within two ROIs of 200-μm square each and the total area of ROIs was 0.08 mm^2^ (see *cell counting* in Materials and Methods). Each time point included 3 animals, except for 3 d, which included 2 animals. All data are presented as mean ± SEM. ^*^P < 0.05 versus control side; ^**^P < 0.01 versus control side; *a*: *P* < 0.05 versus 5 d of the sameside.

We also compared the number of NeuN-ir cells between top and bottom ROIs to examine layer differences in cell density only in the control side. The data were analyzed with a paired *t*-test. These statistical analyses were also conducted using “anovakun version 4.8.2,” an ANOVA function that runs on R software, under R for Windows (version 3.2.2; R Core Team 2018). Statistically significant differences were considered at *P* < 0.05.

## Results

### Gradual Neuronal Decrease and Apoptotic Cell Death after UV Irradiation

We first examined how the UV lesion is established after UV irradiation. Adult Wistar rats were irradiated with UV light (wavelength 365 nm) of total amount of 2.0 mWh through an optic cannula (400 μm in diameter) over the dura mater, as previously described (Nakata et al. 2018). Seven different time points, at which the rats were perfused for histological analyses, were set after the end of the irradiation; 0-h (0 h, less than 15 min), 2 h, 6 h, 12 h, 24 h, 3 d, and 5d.

The UV lesion was not completely established in one day. HE staining revealed that the UV lesion changed its size and shape throughout the 5-d period (Fig. 1*A*). After an inverted bell-shaped lesion appeared at 0 h, an edema enlarged the lesion in the following 24 h. Thereafter, the edema ceased, and small immune cells congregated within the lesion at 3 and 5 d after irradiation. The neuronal cell population (NeuN-immunoreactive [ir] cells, Fig. 1*B*) was not extinct shortly after the end of UV irradiation. Instead, it gradually decreased over 5 d. Neuronal counting within the lesioned area revealed that UV irradiation reduced significantly the total number of neurons in a time-dependent manner (Fig. 1*C*; two-way ANOVA, irradiation: *F*_(1,13)_ = 256.4912, *P* < 0.0001, Time: *F*_(6,13)_ = 11.6477, *P* = 0.0001, UV × Time: *F*_(6,13)_ = 6.3679, *P* = 0.0026). Myelinated neuronal fibers, immunostained with PLP, also degenerated gradually after irradiation (Supplementary Figure S2).

To investigate the mechanism underlying UV-induced cell death after irradiation, we conducted TUNEL assay, which is often used for the detection of apoptotic cell death, to detect DNA fragmentation. TUNEL-positive cells were observed in the UV-irradiated site at all time points within the UV-lesioned area (Fig. 2*A*, 0 h–5 d). However, fewer TUNEL-positive cells were found on the control side (contralateral side of the UV-irradiated side; Fig. 2*A*, control). Moreover, double staining of TUNEL-positive cells with an antibody against NeuN was observed (Fig. 2*B*). These results can be interpreted as indicating that neural degeneration after UV irradiation was accompanied, at least in part, by DNA fragmentation and was possibly due to apoptosis (discussed below). It should be noted that UV irradiation often induced higher background staining (Fig. 2*A*), but this does not necessarily indicate an increased number of TUNEL-positive cells. This may be because UV irradiation made the tissue fragile and rough and altered the binding property of the dye in the UV lesions.

For quantitative analysis, we counted the number of TUNEL-positive and TUNEL-negative cells (i.e., cells stained only with hematoxylin). Note that TUNEL-negative indicates that the DNA fragmentation in the cell was at an undetectable level at that time point. Two-way repeated measures ANOVA revealed that the UV-irradiated side included significantly more TUNEL-positive cells than the control side (Fig. 2*C*, brown bars, UV: *F*_(1,13)_ = 65.1162, *P* < 0.0001), although the TUNEL-positive cell number did not change along the different time points (Time: *F*_(6,13)_ = 0.2886, *P* = 0.9319, n.s., UV × Time: *F*_(6,13)_ = 2.8095, *P* = 0.0559, n.s.). On the other hand, the number of TUNEL-negative cells on the UV-irradiated side changed significantly with time, but it did not in the control side (Fig. 2*C*, blue bars, two-way repeated measures ANOVA, UV: *F*_(1,13)_ = 220.4998, *P* < 0.0001, Time: *F*_(6,13)_ = 3.6560, *P* = 0.0238, UV × Time: *F*_(6,13)_ = 14.9935, *P* < 0.0001). Post hoc analysis with the Benjamini and Hochberg method for *P*-adjustment further revealed that the UV-irradiated side included significantly more TUNEL-positive cells than the control side at 6 h and 5 d (*P* = 0.0231 and 0.0231, respectively) postirradiation. However, the UV-irradiated side included significantly fewer TUNEL-negative cells at 2, 6, 12, and 24 h (*P* = 0.0335, 0.0008, 0.0167, and 0.00651, respectively) postirradiation. Observations suggested that most cells originally located within the lesion area at the UV irradiation site seemed to start the process of cell death during the first 24 h after the end of UV irradiation, although the onset time varied among cells (see Discussion). A drastic increase in TUNEL-negative cells at 3 and 5 d may reflect an immune reaction after the creation of a focal brain lesion.

In addition, the percentage of TUNEL-positive cells was calculated from the cell count data (Fig. 2*D*). Two-way repeated measures ANOVA and post hoc analyses suggested that UV irradiation increased the percentage of TUNEL-positive cells at the time points 6-h postirradiation and later (ANOVA: UV: *F*_(1,13)_ = 191.9875, *P* < 0.0001, Time: *F*_(6,13)_ = 2.4174, *P* = 0.0859, n.s., UV × Time: *F*_(6,13)_ = 8.3031, *P* = 0.0008; UV vs. control, *P* = 0.0033, 0.0317, 0.0091, 0.0317, and 0.0317, respectively; 5 d vs. 6 h or 12 h on the UV side, *P* = 0.0082 and 0.0381, respectively). Taken together, a UV-induced increase of TUNEL-positive cells was observed, especially within 24-h postirradiation. Apoptosis is a strong candidate for the process mediated by UV-induced cellular degeneration. The time course of TUNEL-positivity increase in the present study was parallel to that observed for UV-induced apoptosis in previous studies (reviewed in Kulms and Schwarz 2000).

### Layer-Dependent Difference of Vulnerability to UV Irradiation

**Figure 3:**
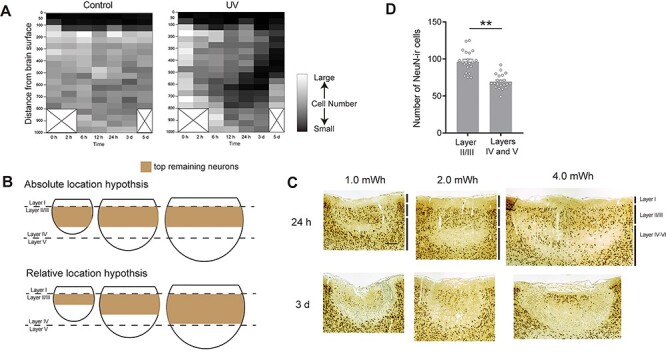
Layer-dependent neuronal degeneration after UV irradiation. (*A*) Heatmaps indicating the topological change of the number of NeuN-positive cells in control (left panel) and UV-irradiated (right panel) sides after UV irradiation. UV-induced neuronal degeneration started from the deep cortical layers within the UV-lesioned area. Cells were counted within an ROI of 200-μm width and 800- or 1000-μm depth located at the center of the UV-lesioned area (see *cell counting* in Materials and Methods). The total cell number was 3781 in the UV side, and 7176 in the control side from 20 animals. (*B*) Schematic illustration of absolute (top panels) and relative (bottom panels) location hypotheses. The brown areas indicate the assumed width of the superficial remaining neurons at 24 h after UV irradiation in each hypothesis. (*C*) Representative photomicrographs of NeuN-immunostained sections in small (created by 1.0 mWh UV irradiation, left panels), medium (created by 2.0 mWh UV irradiation, center panels), and large (created by 4.0 mWh UV irradiation, right panels) at time points of 24 h (top panels) and 3 d (bottom panels) after UV irradiation. This result was replicated 3 times with different animals each time. Scale bar, 200 μm. (*D*) The number of NeuN-positive cells in superficial and deep layers in the control side. The superficial layers (layer II/III) included more NeuN-positive cells than the deep layers (layers IV and V) in the control side. Top (layer II/III) and bottom (layers IV and V) ROIs were 200-μm square each (see *cell counting* in Materials and Methods). Cells were counted in 20 animals. Data are presented as mean ± SEM. ^**^P < 0.01.

Gradual neuronal degeneration indicated that the onset time of the apoptotic process varied among neurons within the lesion. Moreover, it indicated that there were some biological differences in neuronal degeneration (Fig. 1*B*). To evaluate time-dependent topographical change in the neuronal number after UV irradiation, we counted NeuN-ir cells within bins located at a 50-μm distance from the brain surface. The counts revealed that neuronal degeneration within the UV lesion did not occur uniformly (Fig. 3*A*). In the control side, neurons were distributed 150 μm below the brain surface, which was the bottom of layer I that included a few neuronal somata (Fig. 3*A*, left panel). In contrast to the control side, in which there was no remarkable difference in neuronal distribution among seven time points, UV irradiation induced time-dependent reduction in neuronal numbers (indicated by an increase in darker cells of the heatmap; Fig. 3*A*, right panel). Especially at 6, 12, and 24 h, neuronal loss did not progress uniformly in all layers. The bottom part of the lesioned area, located between 500 and 800 μm below the brain surface, included fewer neurons than the superficial layers. It should be noted that, as observed with HE staining (Fig. 1*A*), the edema expanded the lesion area (indicated by smaller cell number in Fig. 3*A*) to the direction of depth at 6, 12, and 24 h, and then, it shrank at 3 and 5 d. Neuronal loss spread within the whole area of lesion at 3 and 5 d. These results suggest that neuronal degeneration in the deep cortical layers progressed more rapidly than in the superficial layers. This is paradoxical because neurons located at the superficial layers have been exposed to larger amounts of UV light; thus, they should have started their degeneration process more rapidly since one would expect that the light should be attenuated during its travel through the brain tissue.

We investigated why this characteristic neurodegenerative pattern (i.e., neuronal loss from the bottom of the lesion) was observed in the UV-lesion method. We hypothesized that the absolute location in the cerebral cortex affects the vulnerability of neurons to UV irradiation. On the other hand, it is also possible that the relative location within the lesion affects the onset time of neuronal degeneration. To examine whether the absolute or relative location determines the neural degeneration pattern in a UV lesion, we modified the lesion size and observed the process of neuronal degeneration after UV irradiation. If the absolute location within the cerebral cortex is important, the remaining neurons should localize at the same location among three lesion areas of different size (Fig. 3*B*, top panels). Alternatively, if the relative location within the UV lesion were the main determinant, the location of the remaining neurons would change depending on the lesion size (Fig. 3*B*, bottom panels). We manipulated the irradiation amount of the lesion size in controls. Irradiation of 1.0- or 4.0-mWh UV light induced smaller or larger lesion than that of 2.0 mWh (medium), respectively. In each group, neuronal degeneration was evaluated at 24- and 3-d postirradiation.

Results revealed that the remaining neurons in layer II/III (top ROI) of a medium size lesion (2.0 mWh) were also observed in both smaller and larger lesions. Moreover, there was no difference in the distribution of remaining neurons in the top ROI among three types of lesion, especially in the direction of depth (Fig. 3*C*). These results support the absolute location hypothesis. Layer II/III was more resistant to UV irradiation than deep layers within the lesion. In addition, in the largest lesion area, some neurons remained in layer V, although most of layer IV neurons disappeared at 24-h postirradiation. Thus, neurons in the upper layers, which were nearer to the UV light source, did not seem to disappear more rapidly than neurons located in lower layers. It should be noted that neuronal density in layer II/III was higher than that in the deep layers (layers IV and V; Fig. 3*D*, *t*_(20)_ = 13.597, *P* < 0.0001, paired *t*-test) in the control side (no UV irradiation). However, neurons in layer II/III were more persistent than deep layer neurons in terms of not only remaining neuronal number but also neuronal survival rate (Supplementary Figure S3). The difference in survival rate suggests that a higher neuronal density may be related to neuronal tolerance in layer II/III. Cortical layer-dependent difference of vulnerability to UV irradiation seems to be a brain-specific feature as a reaction to UV irradiation.

### Glial Congregation and Response to Oxidative Stress after UV Irradiation

**Figure 4:**
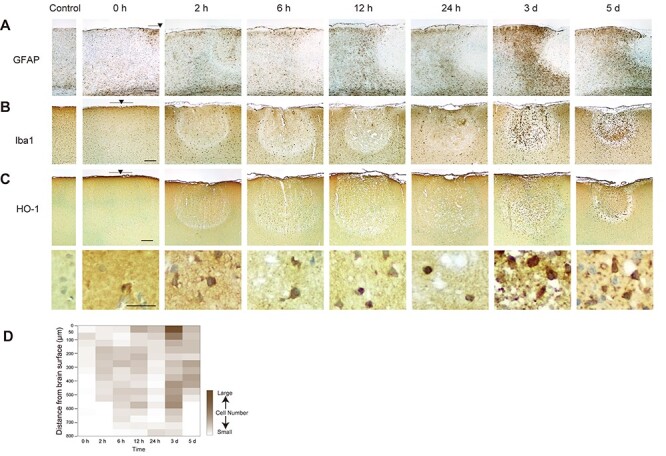
Glial reaction and molecular responses after UV irradiation. Representative photomicrographs of immunohistochemically stained sections with (*A*) GFAP, a marker of astrocytes, (*B*) Iba1, a marker of microglia, and (*C*) HO-1 at each time point after UV irradiation. Sections were counterstained with hematoxylin (blue). Scale bar, 200 μm for (*A*), (*B*), and top panels of (*C*). Scale bar, 20 μm for bottom panels of (*C*). Black arrowhead, center of the UV-lesioned site. Gray bar, coverage area of the core of the optic cannula (400 μm). These results were replicated at least 3 times with different animals each time. (*D*) UV-induced neuronal degeneration started from the deep cortical layers within the UV-lesioned area. The heatmap indicates the topological change of the number of HO-1-positive cells in the UV-irradiated site after UV irradiation. Cells were counted within an ROI of 200-μm width and 800-μm depth located at the center of the UV-lesioned area (see *cell counting* in Materials and Methods).

In addition to neuronal degeneration, we observed the process of glial reactivity to UV irradiation. Generally, focal brain injury induces glial activation and congregation around the lesion site (Norenberg 1994; Streit 2000; Suzuki et al. 2012). To investigate the time course of the glial response after UV irradiation, we examined two types of glial cells: astrocytes and microglia. Immunostaining for GFAP, a marker of astrocytes, revealed that GFAP-ir cells congregated at the center of the UV-irradiated site at 0 h (Fig. 4*A*). These subsequently disappeared at ~6 h. At 3- and 5-d postirradiation, the number of GFAP-ir cells increased around the lesion, an indication of astrogliosis. Immunostaining for Iba1, a marker of microglia/macrophages, revealed that Iba1-ir cells were only sparsely distributed within the lesion up to 12-h postirradiation and subsequently disappeared (Fig. 4*B*). Thereafter, they drastically increased within the UV lesion at 3 and 5 d. These results suggested that glial cells also suffered from UV irradiation and reduced their number, and subsequently, glial congregation started.

In addition to the glial congregation, molecular responses to cellular and tissue damage could have been induced. Among them, HO-1, which is induced responding to oxidative stress, contributes to neuronal protection after brain injury and ischemia by breaking down toxic heme (Maines 1988; Nimura et al. 1996). Immunostaining of HO-1 in UV-irradiated sections revealed that HO-1 was expressed within the UV lesion at all time points examined (Fig. 4*C*). However, almost no HO-1-ir cells were observed in the control side. This indicates that UV irradiation on the brain induces oxidative stress. To evaluate changes in the number and distribution of HO-1-ir cells after UV irradiation, we counted HO-1-ir cells within a 50-μm bin distance from the brain surface (Fig. 4*D*). Topographical analysis further revealed that HO-1-ir cells started to appear in superficial layers (50–100 μm) at 0 h and extended to lower layers. During the first 2 h after irradiation, HO-1 induction was observed predominantly in the upper cortical layers. HO-1 expression expanded throughout the lesion at 6-h postirradiation. It once decreased at 24 h, possibly due to the decrease in total cell number within the lesion; then, it increased drastically at 3- and 5-d postirradiation.

## Discussion

In the present study, we examined temporal changes of cortical tissue after UV irradiation to investigate the process that establishes a UV lesion. UV irradiation induced gradual cellular and tissue degeneration, possibly via apoptosis, during the first 24-h postirradiation. At first, the edge of lesion could be observed in HE stained sections at 0 h, suggesting that the destruction of eosinophilic intercellular tissue had already started, although the number of neurons had not significantly decreased at that time point. Second, degeneration of neurons and other cells became obvious during the first 24 h after the end of UV irradiation. We unexpectedly found that neuronal loss during this period occurred in a layer-dependent manner. Although TUNEL staining revealed that most UV-induced cellular degeneration was accompanied by DNA fragmentation, it still remains to be determined whether UV irradiation just induced apoptosis or UV irradiation directly damaged DNA and then cells degenerated via a process other than apoptosis (e.g., necrosis). Previous studies reported that the induction of apoptosis by UV irradiation at ~8–48 h postirradiation supports the hypothesis that apoptosis is also induced by UV light in the brain (Hseu et al. 2012; reviewed in Kulms and Schwarz 2000). Additionally, it is possible that neuronal death can occur during the irradiation process, although the majority of the neurons died during this 24-h period. Third, activation and congregation of glial cells were observed at 3- and 5-d postirradiation. In addition to cell death, a molecular response to brain injury, including HO-1 expression, was also observed. These results suggest that this UV-lesion method can produce focal brain lesions, which share common processes for the establishment of the lesion and its recovery with those of other types of lesion methods and pathological states (Cortez et al. 1989; Fukuda et al. 1995; Nimura et al. 1996).

The inverted-bell shape of the UV lesion may be due to the optical characteristics of the brain. We examined how UV light diffused in a brain-mimicking optical phantom (modified from Merritt et al. 2003; Hoshino et al. 2004), and found that light diffused in an oval area of 2 mm × 2 mm from the tip of the canula and was drastically attenuated outside of this area (Supplementary Figure S4*A*–*C*). However, when an absorber and a scatter were removed from the brain-mimicking optical phantom, the light diffused widely in a fan-shaped manner from the canula tip (Supplementary Figure S4*D*–*F*). Thus, the optical properties, such as the absorption and scattering of the brain, determine the limited area and the inverted-bell shape of the UV lesion. On the other hand, it would be less likely that UV-induced cell loss was caused by heat damage induced by UV irradiation. We examined whether the temperature increased in a brain-mimicking thermal phantom (Fumoto et al. 2010), whose thermal conductivity of the phantom was similar to that in the brain (0.503 W/[m･K], Olsen et al. 1985). We found that a UV irradiation of 2.0 mWh induced a minimal increase in the temperature of the phantom (Supplementary Figure S5). Taken together, this supported the idea that the focal inverted-bell shape lesion was due to the optical, but not the thermal, properties of the brain.

Among the responses to the UV irradiation, increased expression of HO-1 was a common response to oxidative stress in the damaged brain. Responding to reactive oxygen species (ROS) production, HO-1 is induced following the nuclear translocation of Nrf2, an oxidative stress responsive transcription factor (Ishii et al. 2000; Shih et al. 2003; Hseu et al. 2012). This pathway contributes to neural protection and it is activated after traumatic brain injury (TBI) or ischemia (Fukuda et al. 1995; Nimura et al. 1996). In addition to HO-1 expression, we confirmed nuclear translocation of Nrf2 within the UV lesion (Supplementary Figure S6*A*). In our UV-lesion paradigm, HO-1 seemed to be expressed not only in microglia but also in neurons (Supplementary Figure S6*B*). Although some previous studies reported that HO-1 expression in microglia predominantly responded to brain damage (Ewing and Maines 1993; Matz et al. 1996), it was also reported that the neuronal expression of HO-1 increased after TBI and ischemia (Fukuda et al. 1995; Geddes et al. 1996). Our results suggest that the UV-induced activation of the Nrf2-HO-1 pathway in neurons may be involved in the recovery process although the role of HO-1 in this process from brain injury is still controversial (Beschorner et al. 2000; Wang and Doré 2007). Glial response to UV irradiation also resembles that of other types of brain injury (Cortez et al. 1989; Fukuda et al. 1995; Geddes et al. 1996). Astrocytes were activated at the UV-irradiated lesion site and then disappeared. Thereafter, astrocytes started congregating outside the lesion site and appeared as a scar surrounding the lesioned area as previously described (Burda et al. 2016). On the other hand, the microglia congregated within the lesion at 3- and 5-d postirradiation. Concurrently, the microglia outside the UV-lesioned site extended their processes and somata toward the lesion, indicating microglia migration from the neighboring area toward the lesion (Supplementary Figure S6*C*). At these time points (3 and 5 d), we observed that neuronal loss and tissue destruction progressed more rapidly in a narrow area at the edge of the lesion compared with that in the central area of the lesion. At 3 d, the superficial remaining neurons at the edge side started to disappear, although neurons at the lesion center were still present. This phenomenon was also observed as concentric rings in HE stained samples (3 and 5 d, Fig. 1*A*). It could be that the migrating microglia from outside the lesion edge induced the removal of damaged tissue and cells from the edge of the lesion.

In the present study, we found that the onset time of neuronal loss varied depending on the cortical layer, and that neurons in layer II/III remained longer after UV irradiation than neurons in other deeper layers. Differences in neuronal vulnerability among cortical layers or brain regions have not been studied thoroughly. Although we cannot conclude why the layer II/III of the rat parietal cortex was more tolerant to UV light than other layers, this finding suggests that the time course of lesion creation and recovery may change depending on the subregion of each brain area. In addition to neuronal density (Fig. 3*D*), the distribution of blood vessels varies in a layer-dependent manner (Patel 1983; Huber et al. 2015). According to a previous study, the ratio of vessel surface area to tissue volume was higher in layers where the neuronal soma was dense in the rat cerebral cortex (Hughes and Lantos 1987). Consequently, oxygen supply from the vessels and following ROS generation may be also different among the layers (Masamoto et al. 2004). Previous studies reported that the HO-1 expression level was changed by the oxidative stress level (Hoshida et al. 1996). In fact, our data showed that HO-1 was induced predominantly in the upper cortical layers during the first 2 h after irradiation. Variation in vessel innervation and oxidative stress levels can affect the induction of antioxidant genes, including HO-1, and then induce variation in the time-course of neural degeneration among layers.

UV irradiation also affects the vascular system. We previously reported that UV irradiation above a vessel enlarged lesion size, which supports UV light, can affect the vascular system (Nakata et al. 2018). We also performed supplementary experiments using magnetic resonance imaging (MRI) in mice (Supplementary Figure S7). The mice received i.p. injection of gadolinium hydrate, which cannot travel through the blood–brain barrier. MR imaging showed that the gadolinium had leaked into the brain tissue within the UV lesion at 12–24 h and 5 d after UV irradiation, but not before irradiation (Supplementary Figure S7). This result suggested that UV irradiation damaged the vascular system, although the effect of vascular damage on layer-dependent cellular degeneration should be elucidated in future studies.

Neurons in layer VI, which were located under the bottom edge of the lesion, did not show an obvious decrease with a UV irradiation of 2.0 mWh until 5-d postirradiation. These neurons innervate their fibers toward the superficial layers, including layer II/III (Thomson 2010). Downward destruction and clearance of PLP-ir nerve fibers within the first 24-h postirradiation (i.e., destruction of PLP-ir fibers at the top but not the bottom ROI, Supplementary Figure S2) suggests that UV-induced degeneration of nerve fibers may not directly induce neuronal death. In addition, clearance of axons, but not neuronal soma, at 6-h postirradiation in layers II/III suggests that neuronal fibers were less tolerant to UV irradiation compared with neuronal soma. This finding suggests that the irradiation of UV with an amount below that of the threshold of neuronal loss can induce destruction of nerve fibers.

The UV-lesion method could be clinically applied to stereotactic brain surgery. Localized brain tumors could be good targets of this method. Focal epilepsy may be another target. However, future studies on possible side effects should also be intensely investigated with model animals. In addition, we did not try a wavelength other than 365 nm. Investigating the effect of wavelength would also be important in future studies.

Taken together, we examined how UV lesions were established in the rat cerebral cortex. We observed that UV light induced gradual putative-apoptotic neuronal death, glial congregation, and oxidative stress response during the first 5-d postirradiation. We also found that the time of onset of cell death, revealing the cell’s vulnerability to UV, varied in a layer-dependent manner. Using the UV-lesion method, we can induce a type of stereotypical focal brain lesion in a limited area without dynamic topological change within the lesion. Further work is required to elucidate the mechanism underlying UV-induced cell loss. In addition, the reason why susceptibility is different among different cortical layers and whether this pattern of susceptibility is preserved across regions of the neocortex should be investigated in future studies. Finally, the UV-lesion method may be used as a model of neurodegenerative disorders without obvious tissue loss. The UV-lesion method could also contribute to research about topographical differences in the process of neuronal and tissue degeneration, vulnerability to damage, or the recovery process in the brain.

## Supplementary Material

Nakata_et_al_Supplementary_tgab006

## Notes

The authors thank Yamane S, Miyamoto A, Takashima I, Nagasaka K, Gotoh M, Tachibana Y, and Setogawa T for valuable discussions. *Conflict of Interest*: None declared.
