# Supplementary material for: UV-Induced Neuronal Degeneration in the Rat Cerebral Cortex
Source: Cereb Cortex Commun. 2021 Feb 1;2(1):tgab006. doi: 10.1093/texcom/tgab006 (PMC8152860; doi:10.1093/texcom/tgab006)
Supplement: Nakata_et_al_Supplementary_tgab006 [file nakata_et_al_supplementary_tgab006.pdf]

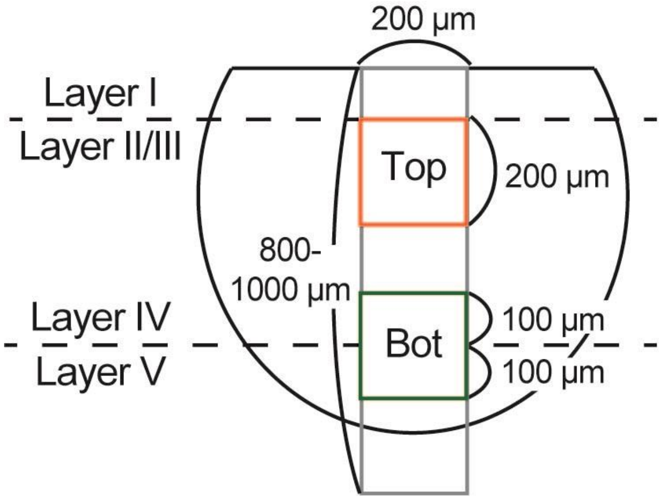

Figure S1. Schematic illustration of the regions of interest (ROIs) used for cell counting.

Large ROI (grey line) was set at the center of the UV lesion and its upper edge was aligned to the cortical surface. The distribution of cells within the large ROI was analyzed. Top (orange) and bottom (Bot) (green) ROIs were set within the large ROI and were 200 µm square each. The number of cells within the top and bottom ROIs were analyzed.

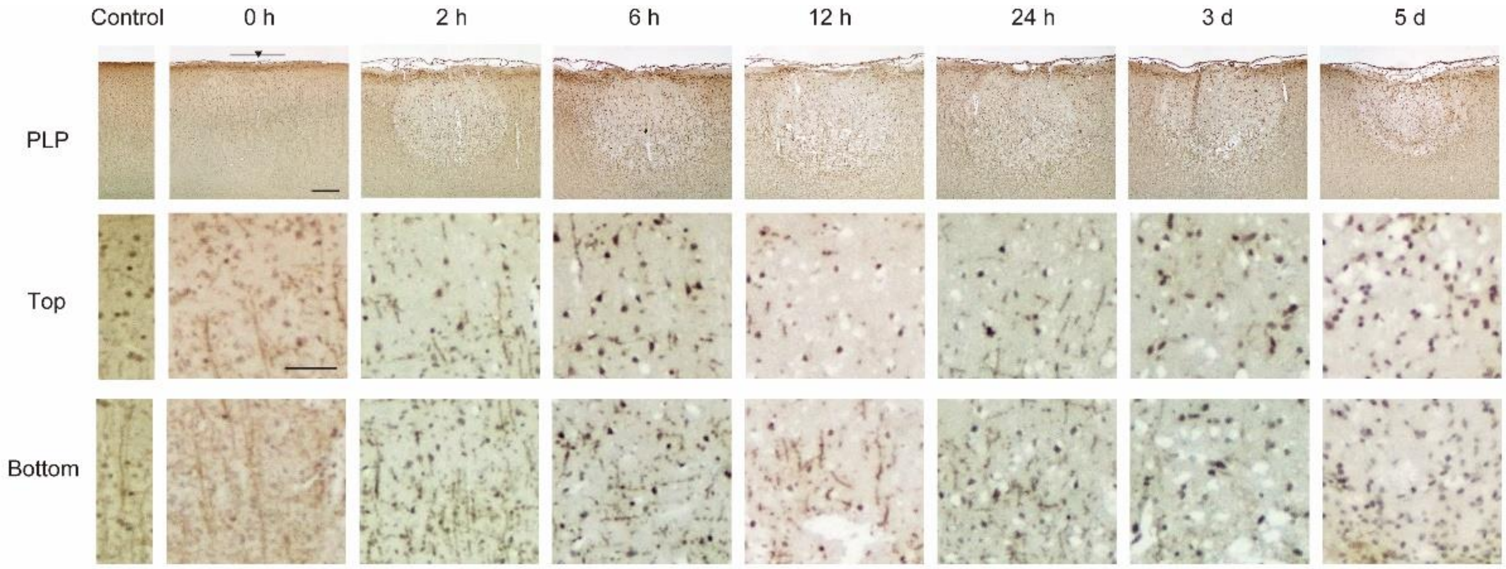

Figure S2. Degeneration process of myelin- proteolipid protein (PLP)-positive fibers after UV irradiation.

Representative photomicrographs of PLP-immunostained sections at each time point after UV irradiation (at bregma -3.84 mm). Brown: Myelin, PLP-positive fibers. Blue: counterstained cells with hematoxylin. Top row: scale bar, 200 µm. Black arrowhead, center of the UV-lesioned site. Gray bar, coverage area of the tip of the optic cannula (400 µm). Middle and bottom rows: within either top or bottom ROI, scale bar, 20 µm.

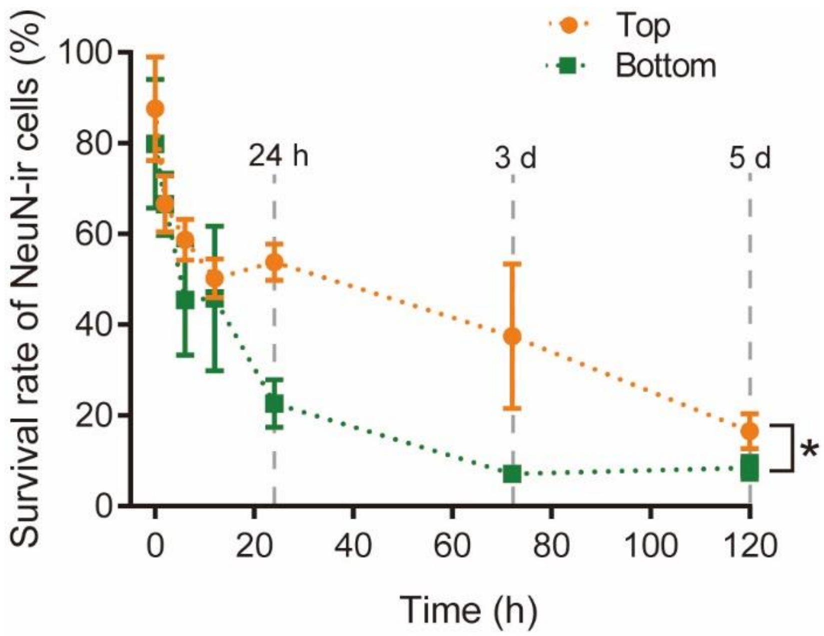

Figure S3. Rapid neuronal degeneration in deep layers after UV irradiation.

Time-dependent change in survival rate of NeuN-immunoreactive (ir) cells after UV irradiation. Neurons in the deep layers (in the top ROI, ●, orange line) decreased more rapidly compared to those in superficial layers (in the bottom ROI, ■, green dashed line; time: $F_{(6,13)} = 13.0886$, $p = 0.0001$, ROI: $F_{(1,13)} = 7.8441$, $p = 0.0150$, time × ROI: n.s., two-way ANOVA, the main effect for time and repeated measurement of ROI, and their interaction). Survival rate = (number of NeuN-ir cells in the UV-irradiated side) / (number of NeuN-ir cells in the control side) × 100. The cell number within the top and bottom ROIs (layer II/III and layers IV and V, respectively) was used for this analysis. All data are presented as mean ± SEM. *: $p < 0.05$; ROI, region of interest; n.s. non significant.

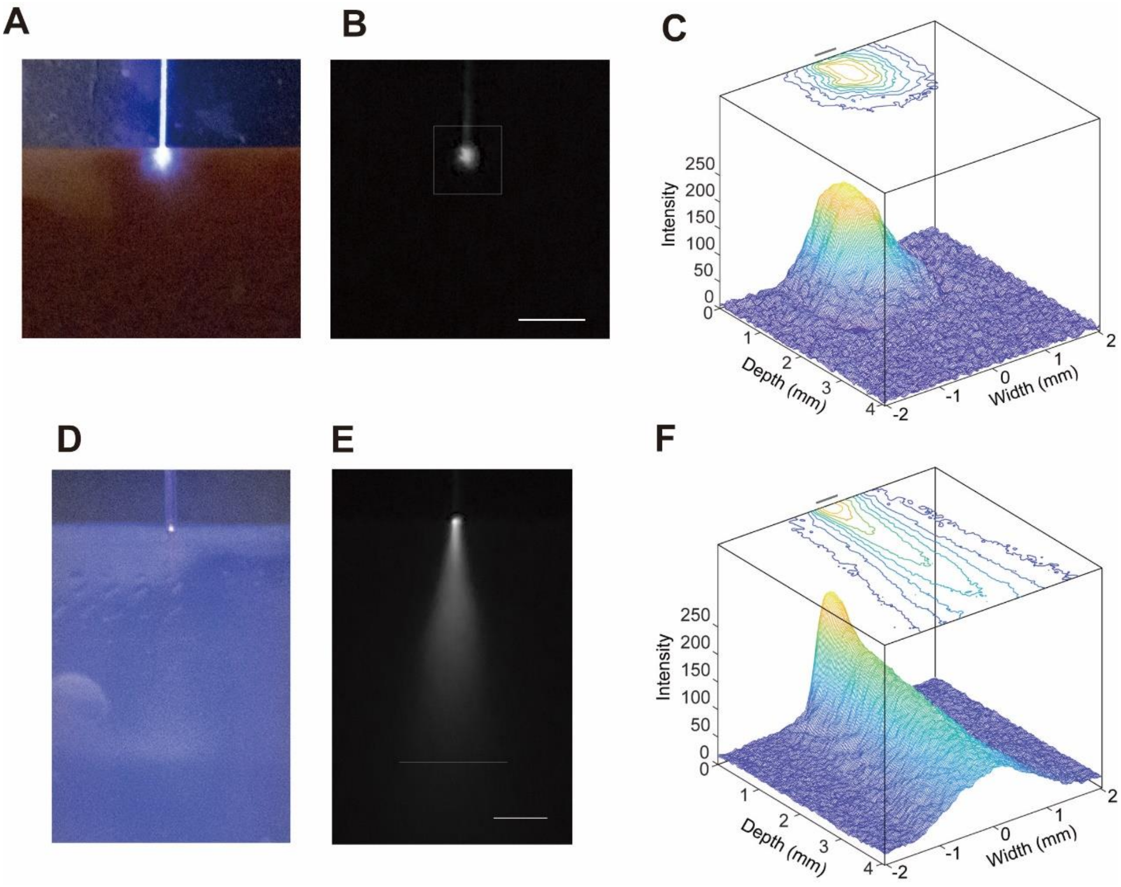

Figure S4. Photographs and intensity map of diffused UV light within a brain-mimicking optical phantom.

To estimate the distribution of the intensity of UV light in the brain, we prepared a brain-mimicking optical phantom based on a previous study (Hoshino et al. 2004). The substrate of the phantom was 0.6% agarose (SeaKem® GTG™ Agarose, Lonza Japan Ltd., Tokyo, Japan) in distilled water, and heparin-added (Heparin sodium injection-N, A Y Pharmaceuticals Co., Ltd.) mouse blood (1.5 volume%) was added as an absorber. As a scatter, 20% intralipid-like solution (20% soy oil in distilled water emulsified by Triton-X 100) was also added (1.0 volume%) (Merritt et al. 2003). The phantoms were then solidified at room temperature. The size of the phantom was 50 × 80 × 15 mm. The optic canula was placed on the edge of the smallest surface, but the entire tip of the canula was on the phantom surface. (A) UV light (1.0 mW; 7.96 mW/mm^2) was irradiated through the optic canula touching the surface of the phantom. (B) UV light diffusion was then photographed with a digital camera (Pentax Q with lens 01 standard prime, RICOH Imaging Company, Ltd., Tokyo, Japan) with a UV permeant filter (UV permeant/visible light absorbing filter U-360, HOYA Corporation, Tokyo,

Japan). The exposure time for UV photography was 30 s. (C) Intensity map. The value of blue color was used as an index of intensity. UV light was diffused within an oval area of 2 mm × 2 mm from the tip of the canula and was drastically attenuated outside of this area. This result is consistent with the inverted-bell shaped UV lesion in the brain. (D-F) To examine how the composition of the brain tissue affects the optical distribution, we also performed the same measurement without the absorber and scatter. Interestingly, the light diffused widely in a fan-shaped manner from the canula tip. These results suggest that the shape of the UV lesion in the brain is determined by the optical characteristics of the brain. (B) and (E): Scale bar = 5 mm. (C) and (F): Gray bar: coverage area of the core of the optic cannula (400 μm).

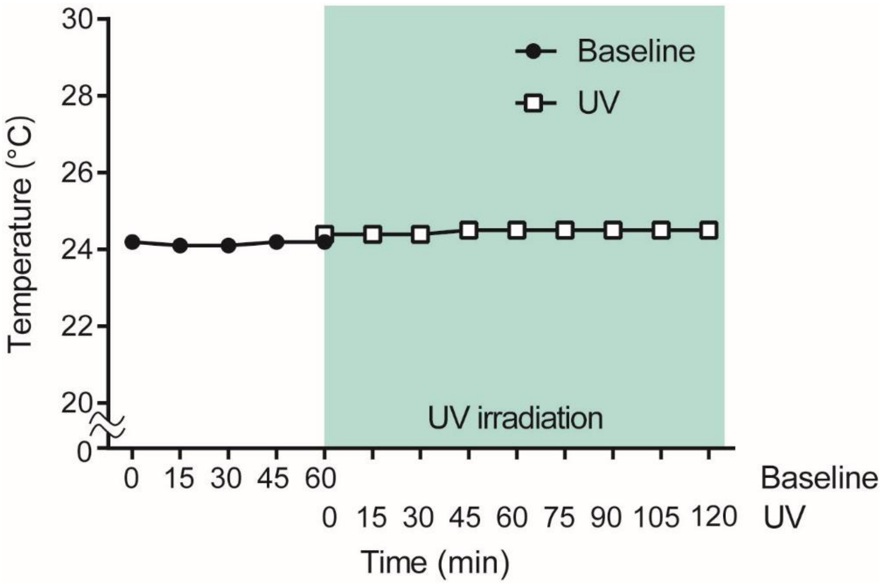

Figure S5. UV irradiation did not induce a temperature increase in a brain-mimicking thermal phantom.

Time-dependent changes in the temperature (°C) of a brain-mimicking thermal phantom before and after UV irradiation. The phantom was prepared from distilled water (95.8 weight%), agarose (3.0 weight%, SeaKem® GTG™ Agarose, Lonza Japan Ltd.), and boric acid (1.2 weight%, KENEI Pharmaceutical Co., Ltd) based on a previous study (Fumoto et al. 2010). They reported that the thermal conductivity of the phantom (agar was used instead of agarose in Fumoto et al. 2010) was 0.53 W/(m·K) and was similar to that in the brain (0.503 W/[m·K], Olsen et al. 1985). A 1.0 mW UV light (7.96 mW/mm^2) was irradiated through an optic canula as described in the Materials and Methods section. The phantom (25 × 18 × 15 mm) was immersed in the water inside a small plastic box (35 × 23 × 23 mm; external dimensions). The box was then embedded within a sponge (45 × 70 × 35 mm) to mimic the rat head. The surface of the phantom was covered with cling film to avoid dehydration. The film was perforated, to allow direct contact of the cannula tip to the phantom. Temperature was recorded with a thermometer (BAT-10R/LOP, Physitemp Instruments, Inc.; Clifton, NJ, USA) by inserting a thermocouple electrode (MT-29/2, Physitemp Instruments, Inc.; Clifton, NJ, USA) every 15 min. The tip of the electrode was stuck beneath the irradiation point 600 µm from the surface. The room temperature was 25.8°C during the experiment. The phantom temperature before UV irradiation was within

the range of 24.1–24.2°C, while that after UV irradiation was within the range of 24.4–24.5°C. This result suggests that the increase in the cortical temperature was minimal.

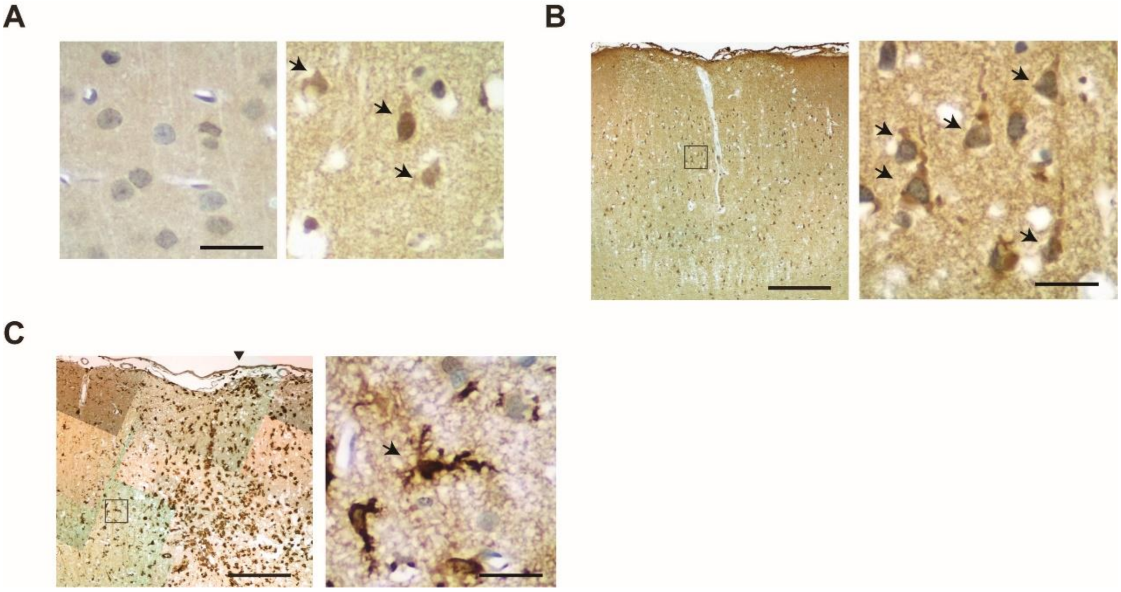

Figure S6. Responses at 6 h after UV irradiation.

(A) NF-E2 related factor 2 (Nrf2) expression in cellular nuclei after UV irradiation. Representative photomicrographs of a Nrf2-immunostained section after UV irradiation (at 6 h). In the control side (left panel), cellular nuclei were stained with hematoxylin, which indicated Nrf2 did not translocate to the nuclei in this area. On the other hand, in the UV-irradiated area (right panel), many cells showed Nrf2-immunostaining (brown) nuclei, which indicated nuclear translocation of Nrf2. Brown: Nrf2-immunoreactivity. Blue: counterstaining with hematoxylin (cellular nuclei). Scale bar, 20 µm, black arrows, cells with Nrf2 translocation into the nucleus. (B) heme oxygenase-1 (HO-1) expression in neurons. Representative photomicrographs of HO-1-immunostained sections after UV irradiation (at 6 h). Pyramidal neurons in layer II/III expressed HO-1 in their cytoplasm. Brown: HO-1-immunoreactivity. Blue: counterstaining with hematoxylin (cellular nuclei). Left panel: scale bar, 200 µm; black square, area indicated in the right panel. Right panel: scale bar, 20 µm; black arrows, HO-1 expressing neurons. (C) Migration of microglia towards the UV lesion. Representative photomicrographs of ionized calcium-binding adapter molecule 1 (Iba1)-immunostained sections after UV irradiation (at 6 h). Activated microglia, immunopositive for Iba1, was located near the UV lesion and migrated towards the lesion. Brown: Iba1-positive cells. Blue: Counterstained cells with hematoxylin. Left panel: scale bar, 200 µm; black square, area indicated in the right panel. Black arrowhead, center of the UV-lesioned site. Right panel: scale bar, 20 µm; black arrow, migrating Iba1-positive cells.

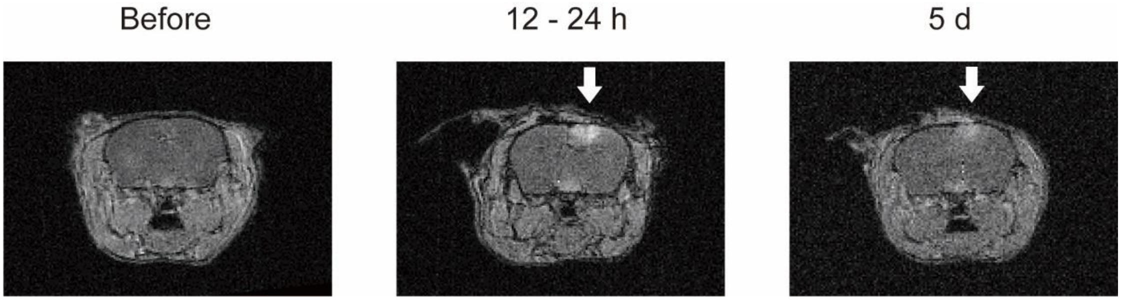

Figure S7. MR images before and after UV irradiation in mice.

MR images of the mouse brain with the blood brain barrier (BBB) impermeant contrast agent gadolinium revealed that BBB leakage was observed after UV irradiation. It is suggested that UV light damages the vascular system. ICR/Jcl adult male mice (n = 3) underwent MR imaging three times: before (left panel), 12–24 h after (middle panel), and 5 d after (right panel) unilateral UV irradiation (2.0 mWh). Thirty minutes before each MR imaging session, mice received intraperitoneal injection of gadodiamide hydrate (10 mmol/kg, OmniScan, intravenous injection, GE Healthcare Pharma, Japan). UV irradiation was conducted using the same procedures as described in the Materials and Methods (but the target site was AP -2.0 ~3.0, ML 1.5~2.5). The mice were anesthetized with 2.0% isoflurane and fixed on a polymethylmethacrylate holder in the prone position. Mice then underwent MRI using an MR scanner (MRmini SA1506, DS Pharma Biomedical Co., Ltd., Osaka, Japan) equipped with a 1.5-T permanent magnet and a solenoid MRI coil (30 mm inner diameter). Coronal MR images were obtained using a T1-weighted three-dimensional fast low-angle shot (3D FLASH) sequence. The imaging parameters for MRI were as follows: repetition time (TR) = 50 ms, echo time (TE) = 4.15 ms (coronal) or 3.6 ms (sagittal), flip angle (FA) = 90°, field of view (FOV) = 20 × 40 × 40 mm, in-plane matrix = 256 × 128, slice thickness = 0.3125 mm (128 coronal slices) or 0.625 mm (64 sagittal slices), and number of excitations (NEX) = 2. As a result, the MR image at 12 – 24 h after UV irradiation showed that the contrast agent leaked out into the brain tissue within the UV lesion. Leakage was still visible at 5 d, although leakage area was reduced compared to that at 12–24 h. This result was replicated three times with different mice. The white arrows indicate the location of UV irradiation.
